# Changing Demographic Profiles of Patients With Traumatic Brain Injury: An Aging Concern

**DOI:** 10.3389/fsurg.2019.00037

**Published:** 2019-07-03

**Authors:** Terence Yi Song Liew, Jun Xuan Ng, Chan Hui Zhen Jayne, Tharun Ragupathi, Colin Kok Ann Teo, Tseng Tsai Yeo

**Affiliations:** ^1^Division of Neurosurgery, University Surgical Cluster, National University Hospital, Singapore, Singapore; ^2^Yong Loo Lin School of Medicine, National University of Singapore, Singapore, Singapore

**Keywords:** demographics, traumatic brain injury, socio-economic burden, elderly, healthcare planning

## Abstract

**Background:** Trauma continues to be a common cause of mortality in Singapore. By understanding the epidemiology of Traumatic Brain Injury (TBI), healthcare professionals can be better equipped to tackle the increasing socioeconomic burden of disease, adopting better strategies in healthcare planning.

**Methodology:** A retrospective review of 367 patients admitted with TBI to a tertiary medical institution from January to December 2014 was performed, studying demographic profiles, injury details and outcomes of these patients. Data was retrieved from the National Trauma Registry and the institution's database.

**Results:** Two hundred thirty-four of the 367 patients included in this study fell into two age groups-−19 to 40 years and ≥65 years. 58% of the TBI population were aged >60. Predominant mechanism of injuries in these groups were road traffic accidents and unwitnessed falls respectively. 39% of the Elderly group were on antiplatelet/anticoagulant agents (*p* < 0.001). While aggressive surgical intervention was more common in younger patients (*p* < 0.001), the elderly group had significantly longer lengths of hospital stay (*p* < 0.001). Though Glasgow Outcome Scale (GOS) scores at discharge were not significantly different between the two groups, elderly patients showed greater percentages of post-injury improvement subsequently.

**Conclusion:** The demographics of TBI patients appears to have shifted toward an older population as compared to a decade ago, with an increased incidence of falls, highlighting a huge healthcare concern. We hope that this study will drive further nationwide studies in future, looking at the incidence and prevalence of TBI, and with the focus on tackling preventable causes of TBI.

## Introduction

Trauma continues to be the fifth most common cause of mortality in Singapore, contributing to 4.7% of all deaths in 2014. Of these patients, traumatic brain injury (TBI) accounts for more than fifty percent of all major disability and mortality (1).

Like most developed countries, Singapore faces the issue of an aging population. The Ministry of Social and Family Development estimates that the percentage of residents aged 65 and over has increased significantly from 8.4% in 2005 to 11.8% in 2015 and is projected to reach 18.7% by 2030.

Given this aging population, one can expect to see certain population trends—increased numbers of TBI due to falls in elderly, (2–11) and among TBI patients, an increased use of antiplatelet and anticoagulation medications. Similarly, patients are more likely to have existing comorbidities such as hypertension, diabetes, and ischemic heart disease, translating to poorer outcomes (2, 5, 10, 12–19). Correspondingly, there will also be limited economic capacity for placements in hospitals.

The management of TBI continues to put an enormous strain on hospital manpower. With (1) the costs incurred both by the hospitals and families involved in patient care, (15, 20–23) and (2) the use of intracranial pressure monitors and extra ventricular drains having risks of device related complications, (24–26) many patients continue to experience effects of TBI long after discharge (2, 3). Studies have shown that the long term life expectancy of TBI patients is lower than the general population, (12, 13, 16) with many of these patients facing chronic disabilities (13, 20, 27). Studies on the association between TBI and dementia have also started to garner public interest (17).

Age has often been identified as a risk factor for poorer outcomes in TBI (3, 5, 8, 12, 15–19, 25, 28). Older patients tend to have poorer outcomes, regardless of other factors such as Glasgow Coma Scale (GCS) score on admission and mechanism of injury. Though these patients tend to have low impact injuries e.g., falls, an increased severity is noted on computed tomography (CT) scans of the brain (7). This could be due to an increased incidence of cerebral atrophy in the elderly population.

This study aims to assess demographic profiles of the local TBI population and serve as a basis for future comparison with local (10) and international data. Examine institutional implications of an aging population on inpatient TBI socioeconomic burden.

## Methods

A retrospective cohort study was carried out on patients with isolated TBI. Patients were identified for the period from 1st January 2014 to 31st December 2014 in a single institution—National University Hospital, Singapore. This data was identified and retrieved from the National Trauma Registry, Singapore, as well as the institution's own patient information database.

For the purpose of our study, patients' TBI severity was classified with the Abbreviated Injury Scale (AIS). The AIS is one of the most commonly used anatomical scales to rate the severity of injuries, including TBIs (29). The AIS can also be used to calculate the Injury Severity Scale (ISS), which is then used to evaluate and prognosticate major trauma or polytrauma. The AIS score ranges from 1, a minor injury, all the way to 6, an unsurvivable injury. Moderate to severe TBI corresponds to an AIS (Head and/or neck) score of 3–5. We also exclude patients with an AIS score of 3 or more in any other body part, as these other injuries may become a potential confounder in assessment of outcomes of TBI. Two major groups of TBI patients were then considered—(1) ages 18–40, representing a majority of Singapore's working class, and (2) ages above 65, corresponding to the retirement age proposed by the Singapore government. This is in contrast to the local study in 2006, which has defined the cut-off at 60 in view of a lower age of retirement at that time.

The data collected includes patient demographics (age, gender, ethnicity, etc), premorbid status (Activities of Daily Living, ambulance, etc), clinical history and findings on admission (mechanism of injury, volume of intracranial bleed, location of brain injury, etc). Glasgow coma scale (GCS) score, presence of skull fractures, midline shift, vital signs, and relevant laboratory investigations. Details of perioperative and postoperative management were collected as well. Indicators of patient outcome considered were length of hospital stay, ventilator dependent days, Glasgow Outcome Scale (GOS), and mortality. Hospital bill amounts were also traced, for each patient, for comparison.

All statistical analysis was carried out using a standardized statistics software. Comparisons between categorical variables of both groups were assessed using Pearson Chi-squared test or Fisher's Exact Test. Continuous variables were assessed using independent *t*-test and ANOVA (normal distribution) or Mann Whitney *U*-test (non-parametric testing).

## Results

Three patients with moderate to severe TBI were admitted to NUH over a 1-year period from January 2014 to December 2014. The demographical data of these patients are summarized in Table 1. There were 250 males and 117 female patients, with ages ranging from 19 to 98 years old. More than half (58.0%) of the cohort was aged above 60. This was significantly higher than historical data, where patients aged above 60 was only reported at 24.6% (10).

**Table 1:** Patient demographics.

	**No**	**%**
**GENDER**		
Male	250	68.1
Female	117	31.9
**RACE**		
Chinese	255	69.5
Malay	42	11.4
Indian	34	9.3
Others	36	9.8
**AGE GROUP**		
0–20	18	4.9
21–40	68	18.5
41–60	68	18.5
>60	213	58.0
**NATIONALITY**		
Singapore	293	79.8
Foreigner	74	20.2
**ADLs**		
Independent	336	91.6
Assisted	21	5.7
Dependent	10	2.7
**AMBULANCE**		
Community	326	88.8
Assisted	28	7.6
Homebound	13	3.5

The details of injury of our patients are summarized in Table 2. While a large proportion of these patients (54.0%) arrived at the Emergency Department with an AIS (Head and Neck) score of 3, there remained a quarter of the patients who arrived with an AIS (H&N) of 5, a critical injury with high risk of mortality. Most patients had favorable GCS scores of 14–15, and/or a mild head injury. The most common mechanism of injury was an unwitnessed fall, corresponding to about half of the total patient cohort. Other common mechanisms of injury included road traffic accidents and witnessed falls. The Activities of Daily Living (ADLs) and ambulance status falls within expected proportions. The observed mortality rate was 8.72%.

**Table 2:** Injury details.

	**Number**	**Percentage (%)**
**AIS H&N**		
3	198	54.0
4	70	19.1
5	99	27.0
**GCS**		
<8	31	8.5
8–13	56	15.3
>13	280	76.3
**MECHANISM OF INJURY**		
Unwitnessed Falls	186	50.7
Witnessed Falls	63	17.2
RTA	69	18.8
Industrial	7	1.9
Fall from height	31	8.5
Assault	11	3.0
**STATUS ON DISCHARGE**		
Alive	335	91.3
Dead	32	8.7

Our cohort was stratified by age into “Young” and “Elderly” groups, summarized in Table 3. Of the 167 patients aged ≥65 years old, 93 were male and 74 were female (range, 65–98 years, 77.6 ± 8.6 years), giving a male-to-female ratio of 4:3. In the younger cohort, aged 19–40 years, the mean age was 30.0 ± 6.3 years, with a male-to-female ratio of 10:1. 62.7% of the “Young” group were foreigners predominantly from neighboring countries. Most held work permits for foreign workers.

**Table 3:** Demographic characteristics.

	**Young (*n* = 67)**	**Elderly (*n* = 167)**	***p*-value**
**Age**	30.0 ± 6.3	77.6 ± 8.6	
Female Gender	6 (9.0%)	74 (44.3%)	<0.001
Race			<0.001
Chinese	24 (35.8%)	141 (84.4%)	
Malay	6 (9.0%)	13 (7.8%)	
Indian	19 (28.4%)	6 (3.6%)	
Others	18 (26.9%)	7 (4.2%)	
Smoking			0.261
Smoker	5 (7.5%)	21 (12.6%)	
Non-Smoker	62 (92.5%)	146 (87.4%)	
Alcohol			0.175
Drinker	2 (3.0%)	13 (7.8%)	
Non-Drinker	65 (97.0%)	154 (92.2%)	
ADLs			0.006
Independent	66 (98.5%)	139 (83.2%)	
Assisted	1 (1.5%)	23 (13.8%)	
Dependent	0 (0.0%)	5 (3.0%)	
Ambulance			<0.001
Community	66 (98.5%)	119 (71.3%)	
Assisted	1 (1.5%)	34 (20.4%)	
Homebound	0 (0.0%)	14 (8.4%)	
Premorbid ASA			<0.001
1	60 (89.6%)	19 (11.4%)	
2	7 (10.5%)	75 (44.9%)	
3	0 (0.0%)	70 (41.9%)	
4	0 (0.0%)	3 (1.8%)	
Anti-coag/platelets		<0.001	
None	67 (100.0%)	102 (61.1%)	
Anticoagulants	0 (0.0%)	12 (7.2%)	
Antiplatelets	0 (0.0%)	52 (31.1%)	
Nationality			<0.001
Singapore	25 (37.3%)	161 (96.4%)	
Foreigner	42 (62.7%)	6 (3.6%)	

Major differences were observed in the mechanisms of injury between the two groups. Unwitnessed falls were the most common mechanism of injury in the elderly group, accounting for 70.7% of injuries. In the young age group, 41.8% were involved in motor vehicle accidents, keeping consistent with developed country data. A significantly greater proportion of females were observed (*P* < 0.001) in the “Elderly” group (44.3%) as compared to the “Young” group (9.0%).

The type of intracranial injury with the highest incidence in both groups was a subdural hemorrhage (SDH), with incidences of 31.3% in “Young” and 58.1% in “Elderly” respectively. However, the “Young” group had an extradural hemorrhage (EDH) incidence of 26.9% while that of the “Elderly” group was 10.8%. Elderly patients were also more prone to multiple bleed locations (66.5%). The incidence of skull fractures in the “Young” group was higher (64.2%) than in the “Elderly” group (29.3%) (*P* < 0.001).

A significant proportion (*P* < 0.001) of the “Elderly” group were on antiplatelet agents (31.1%) or anticoagulants (7.2%), which corresponds with the pre-morbid ASA findings of this group where most had a score of 2 and above (Table 3). The increased use of anticoagulant and antiplatelet agents is made more evident when considering the PTT values and incidence of thrombocytopenia in both groups. Elderly patients tend to be more coagulopathic and thrombocytopenic than their younger counterparts (Table 4).

**Table 4:** Laboratory results.

	**Young (*n* = 67)**	**Elderly (*n* = 167)**	***p*-value**
INR	1.1 ± 0.3	1.2 ± 0.4	0.479
PTT (s)	27.8 ± 3.4	30.0 ± 4.3	<0.001
pH	7.4 ± 0.1	7.4 ± 0.1	0.998
Hb (g/dL)	14.6 ± 1.9	12.3 ± 2.1	<0.001
Thrombocytopenia	1 (1.5%)	23 (13.8%)	0.008
Na (mmol/L)	138.8 ± 16.6	138.0 ± 4.6	0.694

Surgical intervention was more commonly administered in the “Young” group with more than 40% receiving some form of surgical treatment compared to 20% in the “Elderly” group (Table 5). Younger patients also received more aggressive forms of surgical intervention, e.g., craniectomies (*P* < 0.001). This can be explained by the increased SDH incidence in the older group, as most of these patients would receive conservative treatment as compared to the young group where significant proportions had an EDH (Table 6). The shorter time to surgery for the young group can also be explained by the urgency of surgery for EDH as compared to SDH, which are more common in elderly TBI. Elderly patients (24.0%) also received more blood product support than the younger patients (0.0%).

**Table 5:** Surgical intervention.

	**Young (*n* = 67)**	**Elderly (*n* = 167)**	***p*-value**
Whole Blood given	1 (1.5%)	2 (1.2%)	0.856
FFP given	0 (0.0%)	40 (24.0%)	<0.001
Time to Surgery (h)	15.4 (2–144)	52.4 (2–576)	0.156
Surgery Type			<0.001
None	37 (55.2%)	134 (80.2%)	
Craniotomy	1 (1.5%)	0 (0.0%)	
Craniectomy	10 (14.9%)	17 (10.2%)	
Burr Hole	6 (9.0%)	10 (6.0%)	
Others	13 (19.4%)	6 (3.6%)	

**Table 6:** Injury.

	**Young (*n* = 67)**	**Elderly (*n* = 167)**	***p*-value**
Mechanism of injury			<0.001
Unwitnessed fall	5 (7.5%)	118 (70.7%)	
Witnessed Fall	10 (14.9%)	31 (18.6%)	
RTA	28 (41.8%)	12 (7.2%)	
Industrial	4 (6.0%)	0 (0.0%)	
Fall from height	15 (22.4%)	5 (3.0%)	
Assault	5 (7.5%)	1 (0.6%)	
Intracranial Injury			0.001
None	7 (10.5%)	8 (4.8%)	
EDH	18 (26.9%)	18 (10.8%)	
SDH	21 (31.3%)	97 (58.1%)	
SAH	13 (19.4%)	21 (12.6%)	
ICH	6 (9.0%)	22 (13.2%)	
Others	2 (3.0%)	1 (0.6%)	
Location of injury			0.024
None	7 (10.5%)	9 (5.4%)	
Frontal	16 (23.9%)	26 (15.6%)	
Temporal	7 (10.5%)	10 (6.0%)	
Parietal	6 (9.0%)	5 (3.0%)	
Occipital	2 (3.0%)	6 (3.6%)	
Multiple	29 (43.3%)	111 (66.5%)	
Skull Fractures Present	43 (64.2%)	49 (29.3%)	<0.001
Midline shift Present	13 (19.4%)	35 (21.0%)	0.790
Amount of shift	5.6 ± 4.9	8.8 ± 7.2	0.079
AIS (H&N)			0.061
3	43 (64.2%)	80 (47.9%)	
4	12 (17.9%)	37 (22.2%)	
5	12 (17.9%)	50 (29.9%)	
AIS (Others)			
3	0	0	
4	0	0	
5	0	0	
GCS			0.112
Mild	49 (73.1%)	140 (83.8%)	
Moderate	11 (16.4%)	13 (7.8%)	
Severe	7 (10.4%)	14 (8.4%)	
Hypotensive	1 (1.5%)	4 (2.4%)	0.666
Tachycardic	4 (6.0%)	18 (10.8%)	0.255
Tachypneic	14 (20.9%)	19 (11.4%)	0.059
Febrile	0 (0.0%)	7 (4.2%)	0.089
On Oxygen Support	7 (10.5%)	13 (7.8%)	0.510

Comparison of outcomes of TBI between the two groups showed a higher incidence of poorer outcomes in the “Elderly” group (Table 7). This group tended to require much longer lengths of stay (*P* < 0.001). However, there was no significant difference in terms of cost of care between both groups, which is surprising in view of the significant longer length of stay. A larger proportion of elderly patients also had lower GOS scores upon discharge compared to younger patients in the cohort, demonstrating lower rates of functional improvement following TBI. However, subsequent GOS scores for the “Elderly” group revealed higher percentages of post-TBI functional improvement than the “Young” group (*P* < 0.01), suggesting that elderly patients require a longer time to regain normal function.

**Table 7:** Outcomes.

	**Young (*n* = 67)**	**Elderly (*n* = 167)**	***p*-value**
Length of Stay (Days)	8.5 (1–97)	20.4 (1–388)	<0.001
Ventilator dependent days	4.5 (1–8)	6.5 (2–20)	0.679
Readmission	3 (4.5%)	12 (7.2%)	0.445
GOS Discharge			0.030
5	51 (76.1%)	96 (57.5%)	
4	12 (17.9%)	38 (22.8%)	
3	2 (3.0%)	15 (9.0%)	
2	0 (0.0%)	0 (0.0%)	
1	2 (3.0%)	18 (10.8%)	
GOS (3 months)			0.011
5	55 (82.1%)	107 (64.1%)	
4	7 (10.5%)	25 (15.0%)	
3	0 (0.0%)	11 (6.6%)	
2	0 (0.0%)	0 (0.0%)	
1	2 (3.0%)	19 (11.4%)	
Mortality	2 (3.0%)	13 (7.9%)	0.175
Average Hospital Bill Size	8227.0 (422–125,932)	10412.7 (422–164,467)	0.403

## Discussion

### Age Demographics

In this study, the race and nationality are comparable with recently published data on the Singapore population trends. However, in contrast to previous studies done in Singapore in 2006, (10) a significant difference in age demographics was noted. While these studies showed a peak in the 20–40 age group, (10, 30) our study showed a peak in the above 60 age group. Furthermore, while a similar earlier study showed road traffic accidents as the most common cause of TBI locally, (10) our study showed a shift in mechanism of injury to falls, which can be attributed to an increasingly aging population in Singapore.

Nations around the world are facing the crisis of an aging population. Population data from United Nations have shown that the number of persons aged above 60 has increased significantly in many countries, and the rate of growth in number of older persons is expected to accelerate in the coming decades. It is estimated that the number of persons aged above 60 globally is expected to increase from 841 million people in 2013 to above 2 billion in 2050.

Our data showed that older patients tended to be more coagulopathic and thrombocytopenic, given that elderly patients are more prone to diseases such as stroke and malignancies that can cause such bleeding disorders (31). It is thus reasonable to expect to see an increased number of older patients presenting with TBI in the emergency department.

### Falls

Singapore's elderly population is one of the fastest growing in the world, possibly resulting in increasing incidences of fall-related cases seen in local hospitals (32). Though the observed mortality rates of 8.72% were lower than previously reported studies, (33) fall prevention remains critical in preventing such admissions, and will greatly decrease the number of TBI patient admissions. More studies in the community to identify ways to prevent falls in the elderly are warranted. Some commonly used items include grab bars and anti-slip mats in toilets as well as proper lighting in households. Though existing fall prevention programs aim to prevent fragility fractures, the same consideration has yet to be given to head impact (4). Caregiver employment for patients who live alone and ADL dependence can also be explored, since a large proportion of TBI patients suffer unwitnessed falls. Also, there is a trend toward more conservative management for older TBI patients. More studies will need to be conducted to weigh the pros and cons of a surgical approach of treating these patients in view of an increasing lifespan of the general population.

### TBI in Younger Patients

While a larger proportion of TBI patients are older persons, our study still shows a significant proportion of younger patients with TBI. Many of these patients suffer from either a work injury (such as fall from height or industrial accidents) or a road traffic accident. More skull fractures were observed in young patients—this is consistent with the observation that younger patients tended to sustain higher impact injuries like road traffic accidents, especially as pedestrians involved in these accidents (34).

Though much has been done to reduce the incidence and impact of work related injuries, (35) other options can be considered. Foreign workers working here must be provided with medical insurance of adequate coverage based on their industry, and much has already been done to promote helmet use in the construction industry (22). However, there is still a large number of foreign workers suffering from TBI each year. More studies should be done to identify areas for improvement to reduce the incidence of work-related TBI. One example would be working hours, where a study has shown that there were significantly more TBIs at specific times of the day (36).

Road traffic accidents have been and will likely always remain a major cause of moderate to severe TBI. Recently published local data by the Singapore Police Force in 2015 has shown a decline in number of road traffic accidents over the past four years. However, the number of casualties from such accidents still remain high. A developed country study has shown that implementation of motorcycle helmet law results in a lower incidence of TBI due to motorcycle accidents as compared with states in which such laws were not implemented, (37) suggesting that tougher enforcement of seat belt use and laws against drink driving is necessary.

### A Sustainable Healthcare Model

A population report from Singapore Ministry of Health in 2010 identified that falls are the main etiology of TBI among elderly in Singapore. This etiology and its associated outcomes have not changed in 10 years (10, 38). This phenomenon is likely to continue as Singapore's population ages, despite efforts to mitigate falls in elderly.

A 1985 study by Eisenberg et al looked at the changes in the demographic profiles of patients that suffered from Spinal Cord Injuries (SCI) (39). In the study, it has been shown that while more than 70% of patients with SCI fall within the 17–35 age group, it is the long-term SCI survivors, or “veterans,” that require the most long-term care. Eisenberg et al. suggest that more healthcare resources should be channeled to benefit this group of patients.

Based on the epidemiologic transition theory, we are at The Age of Degenerative and Man-Made Diseases (40). This suggests that as science and healthcare progresses, overall mortality will decline and approach stability at a relatively low level (40). This also implies that the average life expectancy will increase, leading to more morbidity (degenerative and man-made diseases).

Given the predictability of TBI incidence in elderly, our research findings may form the basis of a stable healthcare model. The extent of healthcare resource allocation toward the elderly group can be better managed so as to achieve cost-efficiency.

## Strengths and Limitations

The main strengths of this study are the detailed focus on the demographics of patients with TBI, with focus on the mechanisms of injury, treatment and morbidity and mortality rates of different age groups. This study also puts into focus areas of interest in healthcare planning, with highlights on certain preventive measures that can be undertaken to reduce incidence as well as morbidity and mortality of TBI. This study includes all patients admitted for major TBI, thus eliminating selection and inclusion bias. The main limitations of this study will be that this is a single center study, with comparisons to only local data. This study also has a relatively small sample size. Further studies can be performed in future with larger sample sizes for more accurate comparisons.

## Conclusion

TBI in elderly has resulted in a huge socioeconomic burden to society. More can be done to decrease the number of elderly TBI patients. It seems that this problem will only grow in both medical and socioeconomic aspects in years to come (15). As the awareness of TBI in the elderly grows, better ideas, and innovative strategies are necessary to prevent unwitnessed falls in the elderly. This will indirectly translate to a lower healthcare cost and a lower socioeconomic burden of TBI in Singapore. Perhaps with prospective data, a standardized outcome assessment for TBI can be formulated, one that can accurately determine the outcomes of patients when they are first seen in the Emergency Department.

## Data Availability

The raw data supporting the conclusions of this manuscript will be made available by the authors, without undue reservation, to any qualified researcher.

## Ethics Statement

Domain Specific Review Board, Reference number: #2015/01173.

## Author Contributions

All authors listed have made a substantial, direct and intellectual contribution to the work, and approved it for publication.

### Conflict of Interest Statement

The authors declare that the research was conducted in the absence of any commercial or financial relationships that could be construed as a potential conflict of interest.
